# MMP-9 Levels and NaOCl Lavage in Randomized Trial on Direct Pulp Capping

**DOI:** 10.1177/00220345211046874

**Published:** 2021-10-27

**Authors:** N.V. Ballal, H.F. Duncan, D.B. Wiedemeier, N. Rai, P. Jalan, V. Bhat, V.S. Belle, M. Zehnder

**Affiliations:** 1 Department of Conservative Dentistry and Endodontics, Manipal College of Dental Sciences–Manipal, Manipal Academy of Higher Education, Manipal, India; 2 Division of Restorative Dentistry and Periodontology, Dublin Dental University Hospital, Trinity College Dublin, University of Dublin, Dublin, Ireland; 3 Statistical Services, Center of Dental Medicine, University of Zurich, Zurich, Switzerland; 4 Department of Biochemistry, Kasturba Medical College–Manipal, Manipal Academy of Higher Education, Manipal, India; 5 Clinic of Conservative and Preventive Dentistry, University of Zurich, Zurich, Switzerland

**Keywords:** pulpitis, endodontics, biomarkers, caries treatment, matrix metalloproteinases, vital pulp treatment

## Abstract

Outcome expectations of direct pulp capping in carious teeth are obscured by a clinically unknown infiltration and breakdown of the dental pulp tissue. Histologic studies showed that this soft tissue breakdown is related to the innate immune system. We hypothesized 1) that a neutrophil biomarker could predict the outcome of direct pulp capping and 2) that using sodium hypochlorite (NaOCl) as a lavage solution to remove necrotized infected pulp tissue could improve it. In this randomized trial in mature posterior teeth causing no or mild discomfort with carious pulpal exposures, pulpal fluid was collected to assess neutrophil gelatinase (matrix metalloproteinase 9 [MMP-9]) per total protein (TP) levels as a predictive local biomarker. Subsequently, the dentin-pulp wound was randomly washed with a 2.5% NaOCl or a physiologic saline solution (1:1 allocation), capped with mineral trioxide aggregate, and the tooth was immediately restored with a resin-based composite restoration. Ninety-six patients were included, and 84 individuals could be followed up to treatment failure or clinically confirmed pulp survival after a minimum of 1 y. The entire data were fitted to a Cox proportional hazards model to assess the influence of the observational variables MMP-9/TP and discomfort with the randomized lavage treatment on pulp survival. The Kaplan-Meier pulp survival rates after 1 y were 55% for saline and 89% for NaOCl lavage. The inflammatory state of the pulp tissue as reflected by MMP-9/TP levels and NaOCl lavage had a highly significant (*P* < 0.001 and *P* = 0.004, respectively) impact on pulp survival, while mild preoperative discomfort did not. In conclusion, MMP-9/TP showed great promise as a predictive local biomarker, and NaOCl lavage considerably improved the survival time of cariously exposed and directly capped pulps.

## Introduction

Vital pulp treatment can be defined as a range of therapeutic strategies aimed at maintaining the health of all or part of the pulp (European Society of Endodontology 2019). Currently there is no consensus regarding the conservative management of deep caries that radiographically extends close to the pulp space in permanent nonpainful teeth (Careddu et al. 2021). While conservative caries management strategies aimed at avoiding pulpal exposure can have favorable effects on treatment outcome (Bjørndal et al. 2010), complete caries removal and the visual inspection of the pulp under magnification have diagnostic advantages (Lin et al. 2020). A recent position statement by the American Association of Endodontists (2021) recommended complete caries excavation, while the European Society of Endodontology (2019) favored a more conservative approach.

From a scientific perspective, the vital pulp treatment conundrum may be linked to 2 issues. The first relates to the proper assessment of the case that is to be treated and the second to the treatment of the exposed and potentially infected pulp tissue itself. With respect to pulpal diagnosis, it is accepted that in vital teeth that have deep caries and exhibit spontaneous pain, bacteria have entered the pulp tissue. However, pain is unpredictable in response to deep caries, as teeth can become necrotic without any history of pain (Hasler and Mitchell 1970; Michaelson and Holland 2002). Improving pulpal diagnostics and assessment is an area of particular interest in vital pulp treatment—specifically, the discovery of predictors foretelling why teeth with deep or extremely deep caries fail and often at a high rate, even if there are few if any symptoms (Bjørndal et al. 2010; Mejare et al. 2012). Caries is a biofilm-induced disease that activates the pulpal innate immune system in a defensive response to irritation (Meyle et al. 2017). The expression of local biomarkers related to neutrophil presence in the pulp wound presents a potential opportunity to predict treatment outcome (Guthrie et al. 1965; Rechenberg and Zehnder 2014; Mente et al. 2016), as neutrophils are scarcely present in healthy conditions (Nair et al. 2008) and their proteolytic enzymes are the main cause of soft tissue destruction (Cooper et al. 2017). Indeed, neutrophils and their enzymes have been shown to surround microabscesses in the inflamed pulp immunohistochemically (Wahlgren et al. 2002). In this context, complete caries excavation and the exposure of the pulp to collect pulpal fluid (Mente et al. 2016) can be favored over selective caries excavation because the volume of dentinal fluid that can be collected from the dentin is too small to allow proper quantification of target proteins (Ballal et al. 2017). A recent clinical trial highlighted the excellent predictive power of matrix metalloproteinase 9 (MMP-9; neutrophil gelatinase) levels in blood samples collected from the pulp on the treatment success of complete pulpotomy in permanent painful teeth (Sharma et al. 2021).

The therapeutic aspect of vital pulp treatment can also be related to neutrophils and the fact that under deep caries, it is possible that infected microabscesses and necrotic areas have formed in the pulp (Langeland 1987). Sodium hypochlorite (NaOCl) has a unique ability to selectively dissolve necrotized rather than intact/vital soft tissue and not only disinfects but disintegrates biofilms (Zehnder 2006; Tawakoli et al. 2017); as such, it may be a good alternative to physical pulp removal in strategies aimed at preserving pulp vitality. However, vital pulp treatment trials to date have focused on either the best material to use to dress the exposed pulp or the volume of physical pulp tissue removal, in the form of direct pulp capping or partial or full pulp chamber pulpotomy (European Society of Endodontology 2019; Munir et al. 2020). At the same time, potentially more important aspects in the management of this infection at the hard-soft tissue interface, such as the cleansing of the exposed pulp-dentin wound, have not been addressed in comparative studies. Recent clinical trials aimed at other research questions have suggested better outcomes with NaOCl for lavage, as opposed to the saline or other inert solutions preferred in older studies (Munir et al. 2020).

Based on the considerations summarized here, the current randomized controlled trial was conducted in carefully selected asymptomatic or mildly symptomatic cariously exposed adult teeth, and its aim was 2-fold. First, as a surrogate for neutrophil presence (Tsai et al. 2005), the range of MMP-9 per total protein (TP) in pulpal fluid collected after exposure was assessed and tested as a predictive local biomarker for pulp survival after treatment. The presence or absence of mild discomfort prior to treatment was recorded. Second, a randomized treatment step was subsequently performed with lavage of the pulp wound with 2.5% NaOCl or physiologic saline solution. Consequently, this study had an observational aspect (MMP-9/TP and discomfort [yes/no]) and a randomized aspect. The impact of these variables on pulp survival was assessed in a Cox proportional hazards model.

## Materials and Methods

### Study Design

This single-center randomized controlled clinical trial included adults (≥18 y) in parallel experimental groups and had a binary-outcome superiority design. Wound lavage was performed with a 2.5% NaOCl solution (KMC Pharmacy) in the test group and an inert physiologic saline solution (0.9% NaCl; Fresenius Kabi) in the control group. Additionally, 2 observational variables were assessed prior to treatment: MMP-9/TP levels (ng/g) and discomfort (yes/no). The pragmatic trial was carried out in a university and primary care clinic setting.

### Case Selection

**Figure 1.**
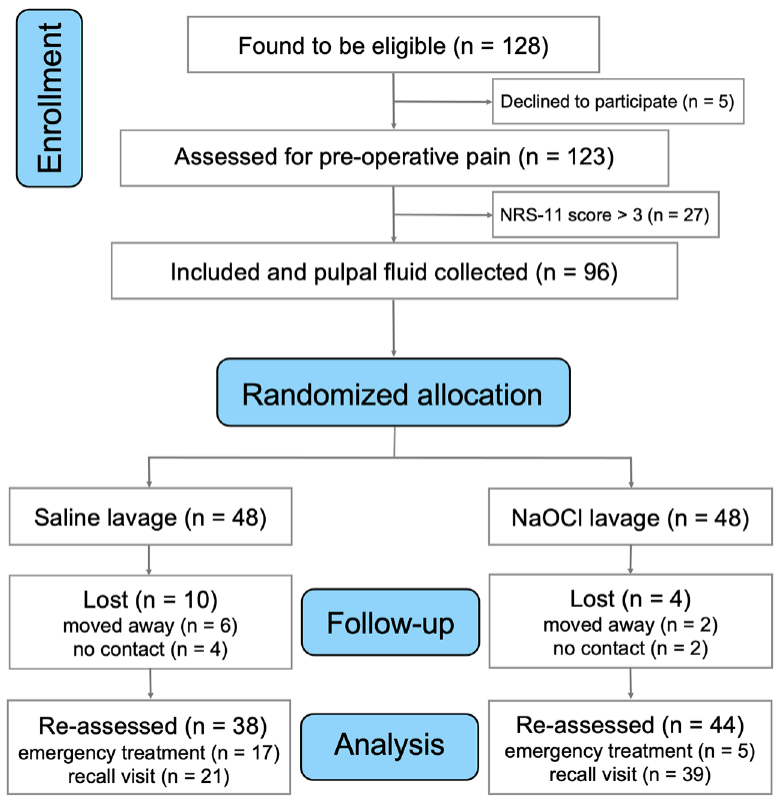
Trial flowchart depicting the journey of the eligible patients from enrollment to final assessment. Eligible individuals were selected from a group of >10,000 patients who were prescreened by the 3 researchers involved in the clinical treatments in the course of their daily clinical duty. NaOCl, sodium hypochlorite; NRS-11, Numerical Rating Scale–11.

Details regarding the recruitment of this strictly defined group of patients and their demography were presented in the preliminary report of this trial, on postoperative pain and early failures, published in an open access communication (Ballal et al. 2020). More than 10,000 patients were prescreened by the 3 researchers involved in the clinical treatments in the course of their daily clinical duty. The trial was approved by the institutional ethics committee (IEC 881/2018) and registered at Clinical Trials Registry–India (CTRI/2019/01/017167). Systemically healthy patients were asked to participate if they met the following inclusion criteria: 1) they presented with caries penetrating the entire thickness of the dentine; 2) pulp exposure was unavoidable (extremely deep caries) after complete excavation in an adult posterior tooth (molar or premolar); and 3) there was no periodontal pocketing and no spontaneous pain emanating from that tooth (for pain, a Numerical Rating Scale–11 [NRS-11] score <4; Ferreira-Valente et al. 2011; Fig. 1). The tooth had to be responsive to a cold test (Endofrost; Coltène) and electric pulp testing within normal limits. Only teeth that did not show any rarefaction on the periapical radiograph were included in the trial. Patients were informed regarding the benefits, risks, and alternative treatment choices before enrollment in the trial. Informed consent was obtained from all patients. The study was conducted in accordance with the guidelines of the World Medical Association Declaration of Helsinki. The CONSORT guidelines for randomized trials were followed.

### Power Analysis and Allocation to Treatment

The sample size was based on the primary outcome, which was pulp survival. Previous prospective clinical trials of over 1-y duration, although not comparing lavage, suggested that caries exposure, washing with NaOCl, and subsequent pulp capping should result in a 90% success rate (Hilton et al. 2013; Hegde et al. 2017; Kundzina et al. 2017). Equivalent carious pulp-capping studies using saline (Bjørndal et al. 2010) or hydrogen peroxide (Barthel et al. 2000) for wound cleansing reported lower pulp survival of 31% and 55%, respectively. These figures, however, may be attributable to other variables, such as the capping material that was used and not simply the lavage employed (Munir et al. 2020). It was thus suggested that 65% was a reasonable success for carious pulp capping at 1 y washed with saline. This represented a clinically important difference of 25% in pulp survival rates between saline and NaOCl pulp lavage, on which the power calculation for this study was based. Therefore, for this binary-outcome, parallel-group superiority trial, a sample size of 40 per group (80 in total) was chosen to achieve 80% power with a type I error of 0.05 and a type II error of 0.2, which resulted in 48 patients in each group (96 total) accounting for a 20% dropout. Forty-eight pulp exposures were to be washed with saline and 48 with 2.5% NaOCl, per a computer-generated number (www.randomizer.org) and block randomization technique (block size of 6). Allocation concealment was achieved through the SNOSE technique (sequentially numbered opaque sealed envelopes) with a 1:1 allocation ratio.

### Clinical Procedures

Local anaesthesia (2% lidocaine hydrochloride with epinephrine, 1:80,000; Septodont) was administered and teeth isolated with a rubber dam (Coltène/Whaledent Inc). All subsequent treatment steps were performed under magnification (EyeMag Smart; Zeiss). Cavities were prepared with a diamond-coated bur (Horico Dental) in a contra-angle handpiece under constant water cooling. The caries was completely (nonselectively) removed from all of the cavity, and excavation continued until the dentin was hard. Bleeding from the exposed pulp was controlled by pressing a cotton pellet soaked in sterile physiologic saline against the wound for 1 min. Subsequently, pulpal fluid was collected with an inverted size 60 sterile paper point (Dentsply; Maillefer) for protein analysis. At this point, one researcher who was not part of this study picked up a closed envelope containing the instructions to hand over 2.5% NaOCl or sterile physiologic saline solution in a glass beaker to the blinded clinician treating the case. The exposure was thus treated for another 30 s with the allocated solution. A cotton pellet soaked in the test or control solution was pressed against the pulp wound, and the cavity was swabbed gently. The cavity was then flushed with sterile saline for 10 s and blotted dry with sterile cotton pellets. Mineral trioxide cement (MTA; Medcem) was mixed according to the manufacturer’s instructions and placed on the pulp exposure site with a small ball-ended carrier (Hu-Friedy). After the initial MTA set, a resin-modified glass ionomer liner (Ionolux; VOCO) was placed over the pulp-capping material. Subsequently, the cavity was etched with phosphoric acid and bonded (Adper Single Bond 2; 3M ESPE), and a posterior resin composite (Filtek Z359 XT; 3M ESPE) was placed incrementally into the cavity and light cured (Elipar LED; 3M ESPE). Finally, the occlusion of the patient was evaluated and corrected if necessary.

### Protein Levels

Paper points (1 per case) were collected in individual sterile microcentrifugation tubes and stored at −80 °C until analysis. On the day of analysis, the samples were eluted in 2 mL of sterile phosphate buffered saline (pH 7.2) by centrifuging at 2,000 × *g* for 30 min at 4 °C. The supernatant was collected and used for analysis. The levels of MMP-9 were measured with a commercially available specific enzyme-linked immunosorbent assay kit (Quantikine ELISA; R&D Systems). The detection range of the assay is 31 to 2,000 pg/mL. MMP levels were normalized to TP in each sample. TP was determined per the Biuret method (Burtis et al. 1999) against a standard series of bovine serum albumin.

### Pulp Survival

Patients were instructed to contact the treating dentist in case of swelling, severe pain, or sensitivity that did not subside from the treated tooth over time. In such cases, root canal treatment was performed, and the time to failure was noted. Patients were subsequently recalled after at least 1 y (Fig. 1). On recall, a detailed clinical examination was performed, including a percussion test, a sensibility test with cold and electric pulp testing, and palpation of soft tissues surrounding the treated teeth. The pain questionnaire (NRS-11) was repeated at this visit. The dentin-pulp complex (i.e., apical periodontal ligament space, periapical pathology, pulpal calcification, and resorption) was assessed through digital planar radiography (VistaScan; Dürr Dental SE). Pulp survival was a composite of clinical and radiographic outcome measures defined as sustained pulp viability and health based on these clinical parameters.

### Data Presentation and Analyses

The observational data considered in this study were discomfort before treatment, categorized into yes (NRS-11 score, 1 to 3) versus no (NRS-11 score, 0), as well as crude MMP-9 (ng/mL) and TP (g/L), which were normalized to MMP-9/TP (ng/g). The target variable, pulp survival, was then modeled with a Cox proportional hazards model, with MMP-9/TP as a continuous explanatory variable and with discomfort (yes/no) and the randomized lavage treatment (NaOCl vs. saline) as categorical explanatory variables. Potential interactions between explanatory variables were investigated by using model comparisons, and eventually the additive model was chosen. The proportional hazards assumption was thoroughly checked by using the test based on weighted residuals and diagnostic plots.

For visualization (e.g., presentation of survival curves) and exploratory purposes, the continuous variable MMP-9/TP was dichotomized with respect to pulp survival rates per receiver operating characteristic analysis and Youden’s *J* as a cutoff point.

Descriptive statistics, statistical models, model diagnostics, and plots were computed with the statistical software R language (R Core Team Version 4.0.2), including the packages survival, survminer, cutpointr, and tidyverse.

## Results

Ninety-six adult patients with extremely deep caries met the inclusion criteria for this direct pulp-capping trial, with 82 followed up to the end point of pain/discomfort and subsequent root canal treatment (*n* = 22) or clinical and radiographic assessment after a minimum of 1 y at recall (*n* = 60). This corresponded to a recall rate of 85.4%. The mean ± SD time from treatment to recall for outcome assessment of the teeth without painful failure was 436 ± 65 d (minimum, 371 d; maximum, 580 d). At the recall visit, only 1 tooth from the control group was nonvital and was added to the failed cases; the remainder (59 cases) were classified as clinically and radiologically successful. More cases failed in the control group, in which only a saline solution was used for wound lavage, as compared with the test group, in which 2.5% NaOCl solution was used. The Kaplan-Meier pulp survival rates were 55% for saline and 89% for NaOCl.

The randomization process resulted in a relatively even distribution of observational values between groups (Table). Two- and 3-way interactions among MMP-9/TP, treatment, and discomfort were tested in the Cox proportional hazards models but had a negligible effect. The observational value MMP-9/TP and the randomized treatment had a highly significant impact on pulp survival (*P* < 0.001 and *P* = 0.004, respectively) while discomfort did not (*P* = 0.227).

**Table.** Distribution of Observational Parameters before Treatment Between the Randomized Groups.

Parameter	Physiologic Saline (*n* = 48)	2.5% NaOCl (*n* = 48)	*P* Value
Discomfort			0.071^a^
Yes	30	38	
No	18	10	
MMP-9, ng/mL	0.8 (2.0)	0.3 (1.4)	0.157^b^
Total protein, g/dL	4.3 (2.1)	5.2 (2.0)	0.038^b^
MMP-9/total protein, ng/g	14.7 (58.8)	6.7 (24.4)	0.072^b^

Values are presented as number or median (interquartile range).

MMP-9, matrix metalloproteinase 9.

a Chi-square test.

b Wilcoxon signed rank test.

**Figure 2.**
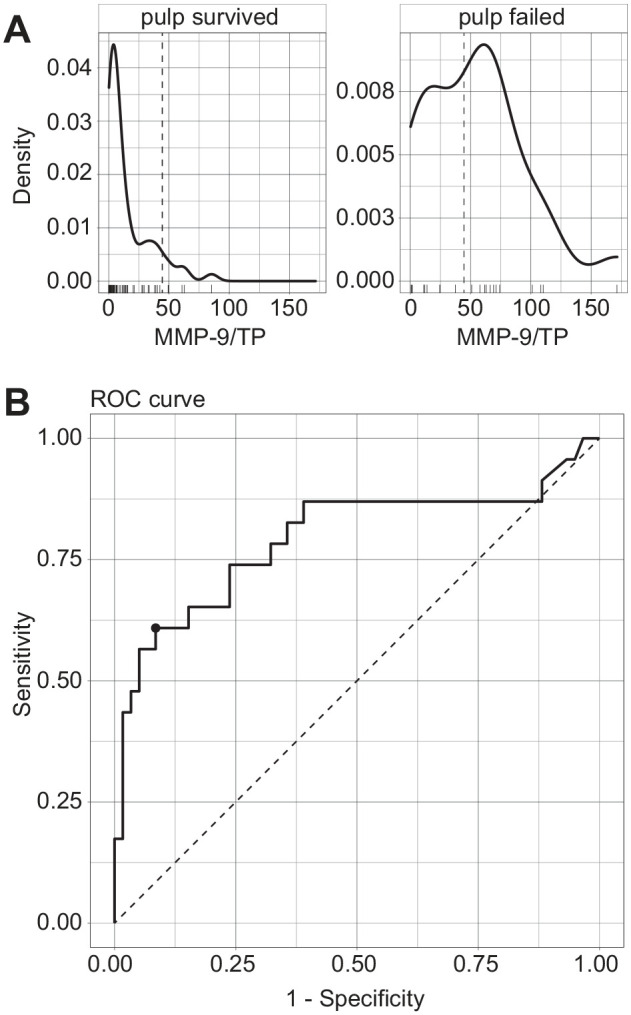
MMP-9/TP variable in view of pulp survival rates. (**A**) Density distribution of MMP-9/TP values (ng/g) in patients whose pulp survived (left) and whose pulp did not survive (right). (**B**) ROC curve depicting the chosen cutoff point for the dichotomization—specifically, the maximum value of Youden’s index, which was determined to be at MMP-9/TP = 44.4 ng/g in our sample. Youden’s index (sensitivity + specificity – 1) is often used as a criterion for dichotomizing numeric values and can be represented graphically as the height of the ROC curve above the dotted chance line. Sensitivity and specificity are shown on a percentage scale (0 = 0%, 1 = 100%). MMP-9, matrix metalloproteinase 9; ROC, receiver operating characteristic; TP, total protein.

**Figure 3.**
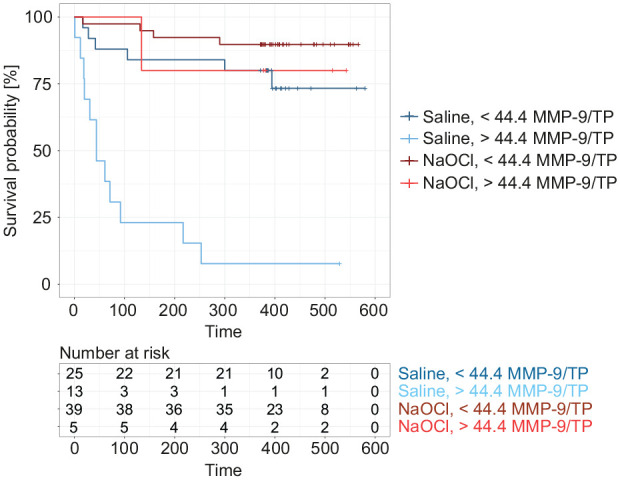
Kaplan-Meier survival curves for 4 subgroups that were treated with saline or NaOCl pulp wash and exhibited initial MMP-9/TP concentrations larger or smaller than 44.4 ng/g. Small vertical dashes show censored observations that could no longer be followed up. Time is shown in days, and the corresponding table shows the number of observations at risk for each time point and subgroup. MMP-9, matrix metalloproteinase 9; NaOCl, sodium hypochlorite; TP, total protein.

The cutoff value for dichotomizing MMP-9/TP in view of pulp survival was 44.4 ng/g per receiver operating characteristic analysis (Fig. 2). With this cutoff value, survival curves could be constructed showing the effect of lavage treatment and initial MMP-9/TP values (Fig. 3). It became apparent that saline lavage of a dentin-pulp wound with high initial MMP-9/TP values led to particularly low pulp survival, while NaOCl lavage could counteract these inflammatory changes.

## Discussion

This study evolved around a lack of accurate diagnostic tools able to delineate the true state of the pulp or identify the threshold among different levels of pulpal inflammation (Mejare et al. 2012). Pulp necrosis progresses from crown to root and is accompanied by the infiltration of the pulp tissue with bacteria, neutrophils, and subsequent microabscess formation (Langeland 1987; Ricucci et al. 2014). Histologically determined “irreversible” pulpitis is a function of neutrophil activity against the invading bacteria (Guthrie et al. 1965; Ricucci et al. 2014), which the current trial considered an opportunity both diagnostically and therapeutically.

A limitation of this study is the low recall rate in view of the relatively short recall time, which is related to the current COVID-19 situation. We will attempt to recall all patients again in 2 y for a 3- to 4-y recall. This was a single-center trial; hence, the described treatment outcomes must be extrapolated to other centers cautiously, as they are potentially linked to operator skill and experience (Munir et al. 2020). Three operators performed the treatments: all were trained endodontists and evenly distributed between test and control treatments (Ballal et al. 2020). A further limitation was that we chose to perform complete rather than selective caries removal in this trial, which can be considered unfashionable from a minimally invasive dentistry perspective (Schwendicke et al. 2016). However, this view is based at least partly on pulp exposure being a negative prognostic factor, a finding that remains far from clear from recent clinical studies (Bogen et al. 2008; Marques et al. 2015). In an earlier investigation examining MMP-9 levels, the MMP-9 collected from the dentinal fluid in the dentin wound was correlated to carious lesion depths within patients (Ballal et al. 2017). That trial revealed that while there were higher levels of MMP-9 in deep as compared with shallow caries lesions, protein yields in dentinal fluid are simply too low to allow proper biomarker quantification and normalization to TP values. Hence, it would appear that the current approach of predictably collecting fluid at least for protein analysis is limited to cases in which the pulp is exposed (Mente et al. 2016).

Studies using a NaOCl lavage on the wound surface, capped with calcium hydroxide or MTA, have reported higher success rates (Hilton et al. 2013; Hegde et al. 2017) than the classic trial of Bjørndal et al. (2010), who used physiologic saline. MTA not only has antimicrobial effects but can induce cell proliferation in injured pulps (Tran et al. 2012). The material was standardized in both arms of the study, and we aimed to examine the additional effect of NaOCl lavage on success. The high efficacy of the 2.5% NaOCl solution in the current trial is most likely linked to the unique feature of NaOCl to selectively dissolve necrotic soft tissue and to its uniqueness among dental antiseptics to dissolve biofilms including their matrix (Zehnder 2006). However, that NaOCl lavage also reduced immediate postoperative pain in this trial (Ballal et al. 2020) was not expected, given the high cytotoxicity of this compound (Aubut et al. 2010). Because of the histologic appearance of the pulp under deep caries with its microabscesses and gradual microbial infiltration in coronoapical direction (Langeland 1987), it would appear that NaOCl lavage in a form of *chemical partial pulpotomy* is a viable option to reduce the necessity to mechanically remove infected tissue. However, further trials and comparative assessments among antiseptics and soft tissue debridement methods are indicated to elucidate this concept. Especially in the context of removing injured pulp tissue mechanically, chlorhexidine may be a better option than immediately applying NaOCl, which could obscure the assessment of bleeding time (Matsuo et al. 1996). The use of chlorhexidine would have the additional advantage of counteracting collagen degradation induced by MMP-9 release (Tjäderhane et al. 1998; Gendron et al. 1999).

Future studies could try to delineate MMP-9/TP cutoff points or the threshold between viable and nonviable tissue damage within a larger cohort of patients with cariously exposed pulps and identical treatments. This could lead to the development of chairside tests, perhaps in the form of rapid membrane-based lateral flow immunoassays (Heikkinen et al. 2016). In the context of minimally invasive dentistry, such tests could be used to determine whether direct pulp capping, full pulp chamber pulpotomy (Sharma et al. 2021), or pulpectomy would be the ideal treatment for the cariously-exposed pulp.

## Author Contributions

N.V. Ballal, contributed to conception, design, and data acquisition, drafted and critically revised the manuscript; H.F. Duncan, M. Zehnder, contributed to conception, design, data analysis, and interpretation, drafted and critically revised the manuscript; D.B. Wiedemeier, contributed to design and data analysis, drafted and critically revised the manuscript; N. Rai, P. Jalan, V. Bhat, contributed to conception and data acquisition, drafted and critically revised the manuscript; V.S. Belle, contributed to conception and data interpretation, drafted and critically revised the manuscript. All authors gave final approval and agree to be accountable for all aspects of the work.
